# Methylation of the *RELA* Gene is Associated with Expression of NF-κB1 in Response to TNF-α in Breast Cancer

**DOI:** 10.3390/molecules24152834

**Published:** 2019-08-04

**Authors:** Young Ju Jeong, Hoon Kyu Oh, Hye Ryeon Choi

**Affiliations:** 1 Department of Surgery, School of Medicine, Catholic University of Daegu, Daegu 42472, Korea; 2 Department of Pathology, School of Medicine, Catholic University of Daegu, Daegu 42472, Korea

**Keywords:** NF-κB, DNA methylation, RELA, inflammation, TNF-α, breast cancer

## Abstract

The nuclear factor (NF)-κB family of transcriptional factors plays a critical role in inflammation, immunoregulation, cell differentiation, and tumorigenesis. This study aims to investigate the role of methylation of genes encoding for the NF-κB family in breast cancer. We analyze the DNA methylation status of the *NFKB1* gene and the *RELA* gene in breast cancer using pyrosequencing. The expression of NF-κB1 and *RELA* proteins is assessed and the level of RNA transcripts in frozen tissue is determined using RT-PCR. There is no statistically significant difference in the methylation status of the *NFKB1* and the *RELA* genes between tumors and normal tissues. The methylation status of the *NFKB1* gene and the *RELA* gene is not significantly associated with the levels of NF-κB1 transcripts in tumor tissues. However, the methylation level of the *RELA* gene is significantly associated with the level of tumor necrosis factor (TNF)-α. In addition, the level of NF-κB1 transcripts was associated with the levels of TNF-α and IL-4. In tumors with positive TNF-α, the increased methylation level of the *RELA* gene is significantly associated with the positive expression of NF-κB1 transcripts. These results demonstrate that the level of the *RELA* gene methylation is related to the levels of NF-κB1 transcripts under the influence of TNF-α. Further study is needed to determine how TNF-α is involved in the methylation of the *RELA* gene and the subsequent expression of NF-κB1.

## 1. Introduction

The nuclear factor (NF)-κB is a transcription factor that plays a critical role in inflammation, immunoregulation, cell differentiation and tumorigenesis [1]. The NF-κB family consists of five subfamily members including p65 (RELA), RelB, c-Rel, p50/p105 (NF-κB1), and p52/p100 (NF-κB2). These members form homodimers or heterodimers to activate transcription of the target genes, and play an important role through two different signaling pathways, the canonical and the non-canonical pathway [1]. Among the NF-κB dimers, RELA*,* and the p50 heterodimeric complex are the most commonly activated forms in human epithelial cells.

The NF-κB signaling pathway is essential for control of cellular and physiologic functions [1,2]. Activity of the NF-κB is regulated by many divergent stimuli and inhibitory factors [3,4]. The inhibition of NF-κB proteins (IκB) is one of these factors, and these proteins hold the NF-κB dimers in inactive conformations by binding [1]. Conversely, the IκB kinase (IKK) activates the NF-κB by phosphorylating the IκB. Dysregulated NF-κB activity leads to various inflammatory diseases and cancer. In recent years, accumulating data have revealed link between the NF-κB and development and progression of cancer [3,4,5,6]. The NF-κB may be involved in carcinogenesis by mediating inflammation and various oncogenic mutations and contribute to the pro-tumorigenic microenvironment [6]. In addition, other possible roles of the NF-κB in cancer cells include stimulating cell proliferation and preventing apoptosis, regulating tumor angiogenesis, promoting tumor metastasis, and remodeling tumor metabolism [6].

Although the mechanism of the dysregulation of NF-κB activity is not fully understood, several mechanisms have been identified. For instance, abnormal function of the IκB [7], aberrant IKK activity and its subsequent degradations [8] are well-known uncontrolled mechanisms. Recently, lysine methylation of the NF-κB has emerged as an important regulatory mechanism of NF-κB activity [9,10,11]. Several oncogenes and proteins were also reported to be involved in regulating NF-κB activation [4]. In addition, a recent study reported altered DNA methylation and overexpression of the NF-κB-related genes in celiac disease [12]. DNA methylation is different from lysine methylation which is one of the post-translational modifications. DNA methylation of the NF-κB-related genes could affect the expression and the activity of the NF-κB, but there are limited data on gene mutations or altered expressions of NF-κB-related genes in cancer.

Recently, several studies described how aberrant NF-κB activation is associated with breast cancer development and progression [13,14,15,16,17,18]. In addition, increased expression of the NF-κB was detected in breast cancer [13], but the mechanisms regulating the NF-κB expression is still unclear. Yamamoto et al. [16] showed that enhanced NF-κB-inducing kinase (NIK) expression, which is related to activation of the NF-κB dimer in non-canonical pathway, is caused by epigenetic alteration of the *NIK* gene. Likewise, epigenetic mechanisms may be related to the expression of the NF-κB in breast cancer. However, there is no report on the DNA methylation status of genes encoding for the NF-κB family in breast cancer yet. In this study, we hypothesize that the NF-κB-related genes may be hypomethylated in breast cancer. To determine epigenetic alterations of the genes encoding for the NF-κB family, we aim to investigate the methylation status of the NF-κB-related genes in breast cancer. In addition, we analyze the association between methylation of these genes and their expression.

## 2. Results

### 2.1. Methylation Status of the NF-κB-Related Genes in Breast Cancer

**Figure 1:**
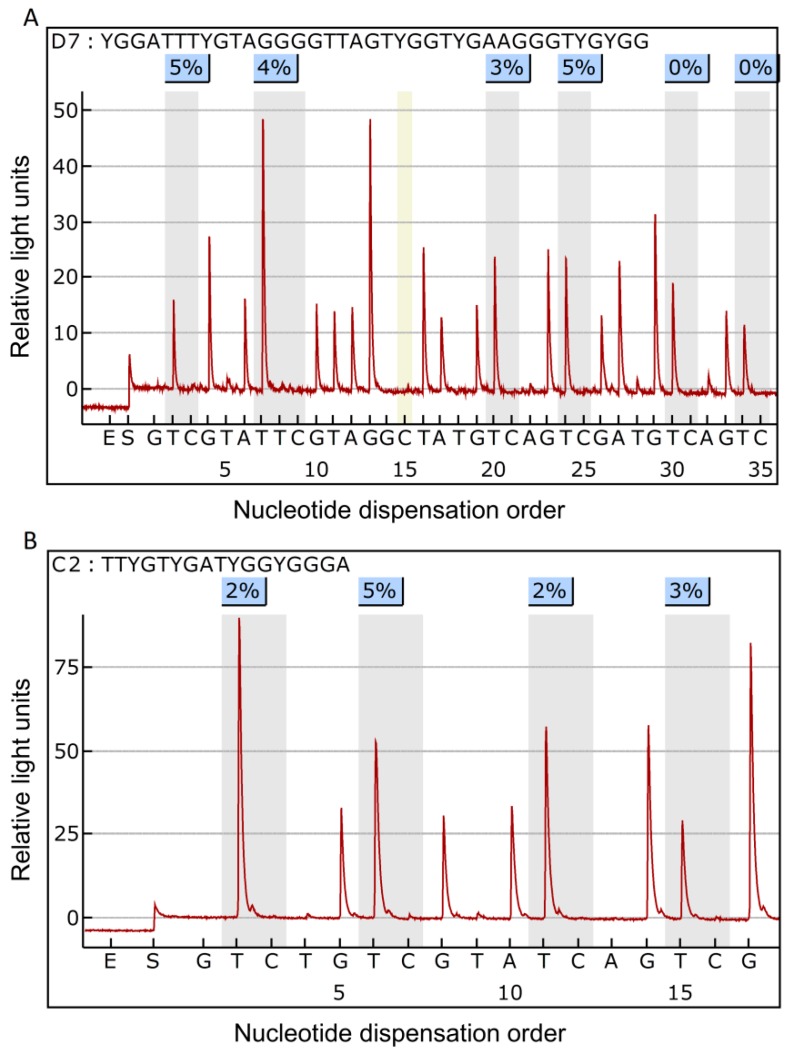
Representative pyrogram of *NFKB1* gene (**A**) and *RELA* gene (**B**). The sequence at the top of the pyrogram represents the sequence to be analyzed. Gray shadowing highlights the analyzed CpG sites. By pyrosequencing, unmethylated cytosine (**C**) is measured as the relative content of thymine (T) and methylated C is measured as the relative content of C at the CpG site. Accordingly, the T and C peaks indicate unmethylated and methylated C, respectively. Percentage values over the C base are the percent of gene methylation at each site which is defined as the percent of C base. The yellow area indicates the portion of the C added to verify complete conversion of unmethylated C to T.

**Figure 2:**
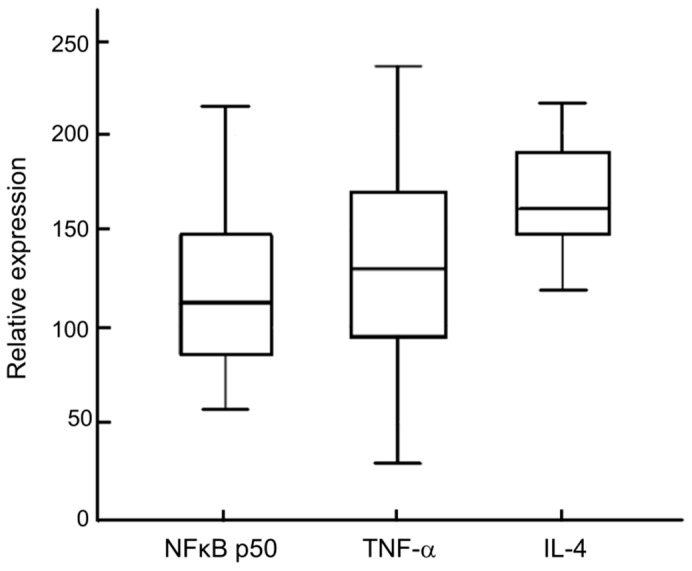
Levels of mRNA transcripts in tumor tissues. The boxes represent the lower and upper quartile limits and the line in the box represents median values for NF-κB p50, the tumor necrosis factor (TNF)-α, and IL-4 mRNA in tumor tissues from breast cancer patients. The bars below and above the boxes show the minimum and maximum expression levels, respectively. Whiskers represent 95% confidence intervals.

In primary breast cancer tissues, the mean methylation level was 1.40 ± 0.90% for the *NFKB1* gene and 1.49 ± 0.50% for the *RELA* gene, while that of two genes in normal mammary tissues was 1.51 ± 0.66% and 1.60 ± 0.19%, respectively. There was no statistically significant difference in the methylation status of the *NFKB1* and the *RELA* genes between tumors and normal tissues. Figure 1 shows the representative pyrogram of the *NFKB1* and the *RELA* genes. In the analysis of the association between methylation of these two genes and their expression, the methylation status of the two genes was not related to the level of NF-κB p50 transcripts in tumor tissues (*p =* 0.717 and *p* = 0.182, respectively) (Table 1). Figure 2 shows levels of mRNA transcripts of NF-κB p50, tumor necrosis factor (TNF)-α, and interleukin (IL)-4.

### 2.2. Association of Methylation Levels of the NF-κB-Related Genes and Gene Expression with Inflammatory Markers in Tumor Tissues

The NF-κB signaling pathway is related to the inflammatory response [2] and may contribute to carcinogenesis [3]. We compared the methylation status of the two genes and the levels of inflammatory cytokines in tumor tissues to analyze the association between methylation of the NF-κB-related genes and inflammation in breast cancer. The mean methylation level of the *RELA* gene was significantly associated with the level of TNF-α (*p* = 0.009) (Table 2). In addition, the level of NF-κB1 transcripts was associated with the level of TNF-α and IL-4 (*p =* 0.001 and *p* = 0.014, respectively) (Table 3). The level of TNF-α was significantly higher in tumor tissues than in normal tissues (*p* = 0.006). To investigate the role of TNF-α, we analyzed the association of the level of NF-κB p50 transcripts with the methylation status of the *NFKB1* and the *RELA* genes in tumor tissues depending on the expression of TNF-α. In tumors with positive TNF-α, the increased methylation level of the *RELA* gene was significantly associated with the positive expression of NF-κB1 transcripts (*p* = 0.002) (Table 1). 

### 2.3. Association of Methylation Levels of the NF-κB-Related Genes with the Clinicopathologic Characteristics

We analyzed the clinicopathologic features of patients to determine the factors involved in methylation of the NF-κB-related genes. There were no clinicopathologic factors associated with the methylation status of the *NFKB1* and the *RELA* genes (Table 4). Additionally, no clinicopathologic factors were associated with the level of NF-κB p50 transcripts in tumor tissues.

## 3. Discussion

In this study, we analyzed the methylation status of the *NFKB1* and the *RELA* gene in breast cancer, and found that the methylation level of the *RELA* gene was significantly correlated with the level of NF-κB1 transcripts under the influence of TNF-α. In addition, the mean methylation level of the *RELA* gene and the level of NF-κB1 transcripts were significantly related to the level of TNF-α. As far as we know, this is the first study investigating whether DNA methylation changes in the NF-κB-related genes occur in breast cancer and affect the expression of the NF-κB family.

DNA methylation is a well-known epigenetic modification of genes and is important in regulating gene expression and functioning in normal cells. Methylation of CpG islands results in gene silencing and contributes to the inhibition of gene expression [19]. DNA hypermethylation of certain tumor-suppressor genes is significantly linked to cancers, while hypomethylation of several genes are known to be involved in chronic inflammation and some cancers [20]. For decades, the NF-κB has been known to be involved in inflammation and carcinogenesis. A previous study investigated the methylation status of the *NFKB1* and the *NFKB2* gene as a measure of inflammation based on the fact that the *NFKB* genes play an important role in inflammation and are suggested to be deactivated by DNA methylation [13]. The authors showed that hypomethylation of the *NFKB* genes after exercise training tends to promote inflammation in older people [13]. Recently, Fernandez-Jimenez et al. [12] analyzed DNA methylation changes of eight NF-κB-related genes in celiac disease, and showed that methylation level of the *RELA* gene was lower in celiac disease than non-celiac controls. In this regard, several genes involved in the NF-κB signaling pathway may be hypomethylated in inflammation. In the same way, we hypothesized that the NF-κB-related genes could be hypomethylated in breast cancer based on the fact that increased activity of the NF-κB is detected in breast cancer [13]. In our study, mean methylation frequency of the *NFKB1* and the *RELA* gene in breast cancer was relatively lower than those of normal mammary tissues, but there was no statistically significant difference. As Fernandez-Jimenez et al. [12] showed that methylation levels of the NF-κB-related genes varied depending on the disease status, methylation status of the NF-κB-related genes in breast cancer may depend on the tumor microenvironment. Although normal mammary tissues in our study were distinct from the tumor, these control tissues may have similar tumor microenvironments because they were taken from the breast specimens around the tumor, which may have affected the results. Normal breast tissues from healthy women may be needed for better control.

One of the most important findings of this study is that the methylation level of the *RELA* gene showed positive correlation with NF-κB1 transcripts depending on expression of TNF-α in tumor tissues, whereas the methylation status of the *NFKB1* and the *RELA* genes were not directly associated with the level of NF-κB p50 transcripts. In view of the importance of the role of TNF-α in the NF-κB pathway, TNF-α may involve in the methylation of the NF-κB-related genes and subsequent expression of these genes. Our findings, which show the association between the *RELA* gene methylation and TNF-α, support this notion. Furthermore, in consistent with previous studies [21,22], our study showed that high level of NF-κB1 transcripts correlated with positive expression TNF-α. It is well-known that TNF-α interacts with the NF-κB through various mechanisms. TNF-α induces activation of the NF-κB pathway [21,22,23]. Conversely, the NF-κB regulates pro-inflammatory cytokines such as TNF-α and contributes to the pro-tumorigenic microenvironment [6]. Recently, TNF-α has been suggested to regulate certain gene expressions through DNA methylation [24,25]. Acharyya et al. [24] reported that TNF-α promotes DNA methylation at the *Notch-1* promoter in skeletal muscle cells by recruitment of histone and DNA methyltransferases (DNMT). On the other hand, Morisawa et al. [25] showed that TNF-α increased the levels of DNA methylation, but DNMT did not participate in TNF-α-induced DNA methylation at the *EC-SOD* promoter region in human dermal fibroblasts. Although how TNF-α is involved in the methylation of the *RELA* gene and subsequent expression of the NF-κB1 is not yet clear, there are several possible mechanisms such as regulation of DNMT1 expression by TNF-α [25], epigenetic feedback regulation [26,27], or DNA co-methylation between gene pairs [28]. Because data on the DNA methylation status of NF-κB-related genes are still lacking, additional studies are needed to determine the DNA methylation status of the NF-κB-related genes in breast cancer. Laboratory research is also required to reveal related mechanisms. Our study has several limitations. First, our study includes a relatively small sample size. Further studies with larger numbers of samples are needed to clarify the role of methylation of the NF-κB-related genes in breast cancer. Second, we did not include the status of the *RELA* gene transcript expression by RT-PCR. We have tried several times to get results under different conditions, but failed to produce results due to failed PCR reactions. Further study is required to analyze the expression of the *RELA* gene using other methods. Third, we have not studied specific mechanisms related to our results, which limit understanding of the results. We will continue to study to determine the mechanisms related to our findings, including analysis of the expression status of some downstream genes of the inflammation-induced NF-κB pathway.

In conclusion, we found that the level of the *RELA* gene methylation was significantly related to the levels of NF-κB1 transcripts in breast cancer with positive expression of TNF-α. In addition, the mean methylation level of the *RELA* gene and the level of NF-κB1 transcripts were significantly associated with the level of TNF-α. Our results suggest that methylation of the *RELA* gene contributes to expression of NF-κB1 in response to TNF-α in breast cancer. Additionally, TNF-α may affect the methylation of the NF-κB-related genes and the expression of NF-κB family. Further study is needed to determine how TNF-α is involved in the methylation of the *RELA* gene and the subsequent expression of NF-κB1.

## 4. Materials and Methods

Tumor tissues were obtained from patients who underwent surgery for treatment of primary breast cancer between 2008 and 2012 at Daegu Catholic University Hospital (Daegu, Korea). All specimens were examined by an experienced pathologist and confirmed as invasive ductal carcinoma. Immediately after resection of the tumor from the patients, a small piece of the tumor tissue was collected in a sterile collection tube and a total of 47 tumor tissues were included in the study. For controlled analysis, 10 normal mammary tissues were collected from resected breast specimens separately from the tumor. The samples were stored at −80 °C until further analysis. The remaining tissues were fixed in formalin and embedded in paraffin (FFPE), then stained with hematoxylin and eosin (H & E) for histologic examination. Immunohistochemical staining for estrogen receptors, progesterone receptors, human epidermal growth factor receptor 2, and Ki-67 were performed on FFPE tissues according to the methods described in our previous study [29]. Intratumoral or peritumoral lymphocyte infiltration was also assessed semiquantitatively. Clinicopathologic characteristics of the patients were reviewed from the medical records and pathologic reports. The ethics review of the study was waived from the Institutional Review Board at the Daegu Catholic University Hospital according to the deliberation criteria.

We analyzed the DNA methylation status of the *NFKB1* gene encoding for the NF-κB p105 protein which can undergo cotranslational processing to produce the p50 protein and the *RELA* gene encoding for RELA protein. DNA was extracted from freshly frozen primary breast cancer tissues and isolated using the QIAamp DNA Mini Kit (Qiagen, Hilden, Germany) following the manufacturer’s instructions. Quality control of the purified genomic DNA was checked using a NanoDrop spectrophotometer (Thermo Scientific, Wilmington, DE, USA). A total of 300 ng of genomic DNA was processed for bisulfite treatment and conversion using the EZ DNA Methylation-Lightning kit (Zymo Research, Orange, CA, USA) according to the manufacturer’s instructions. Bisulfite-treated DNA was amplified by polymerase chain reaction (PCR) using a PCR premix (Enzynomics, Daejeon, Korea) with a PCR instrument (Applied Biosystems, Carlsbad, CA, USA) and confirmed by agarose gel electrophoresis. PCR conditions were as follows: 95 °C for 10 min, then 45 cycles of 95 °C for 30 sec, 54 °C~56 °C for 30 sec and 72 °C for 30 sec, and then a final extension of 5 min at 72 °C. The annealing temperature was 54 °C for the *NFKB1* gene and 56 °C for the *RELA* gene. Forward primer for the *NFKB1* gene was 5′-GTTGAGAGGTATATGGGATTAG-3′ and reverse primer was 5′-CACTCCAACCTTCTCACCAT-3′. For the *RELA* gene, forward primer was 5′-GATTGGGGTGGGTAGGATA-3′ and reverse primer was 5′-AATTTCAAAACCCCCTCC-3′. Pyrosequencing was performed for DNA methylation analysis using the Pyro Gold kit and Pyromark ID system (Qiagen, Hilden, Germany) according to the manufacturer’s instructions. For pyrosequencing, the primer was 5′-GATAGAGGGGAAAGATATATT-3′ for the *NFKB1* gene, and 5′-GGTTTAGAGTGATATTATTAAAT-3′ for the *RELA* gene.

To analyze expression of the *NFKB1* gene and the *RELA* gene, the levels of ribonucleic acid (RNA) transcripts in frozen tumor tissues were determined using reverse transcriptase-PCR (RT-PCR. Total RNA was extracted using Trizol reagent (#A33250; Invitrogen; Thermo Fisher Scientific, Inc., Wilmington, DE, USA) according to the manufacturer’s protocol. Reverse transcription was carried out using Superscript II RNase H-reverse transcriptase (#18064071; Invitrogen; Thermo Fisher Scientific, Inc., Wilmington, DE, USA). Also, the level of RNA transcripts of cytokines including TNF-α, IL-2, IL-4, IL-6 and interferon (IFN)-γ was analyzed using RT-PCR in frozen tumor tissues.

Statistical analysis was performed to analyze the association between the methylation status of the genes and the level of RNA transcripts. Association between two categorical variables was analyzed using the Chi-square test or the Fisher’s exact test. To analyze continuous data, two sample t-tests or the non-parametric Mann-Whitney U tests were used. The relationship between clinicopathologic characteristics of the patients and the methylation status of the genes was also analyzed. SPSS software version 19.0 (SPSS, Inc., Chicago, IL, USA) was used for statistical analysis. All tests were 2-sided and a *p*-value of <0.05 was considered to indicate a statistically significant difference.

## Figures and Tables

**Table 1:** Association of methylation levels of the *NFKB1* gene and the *RELA* gene with nuclear factor (NF)-κB p50 transcript in tumor tissues (*n* = 47).

		*NFKB1* Methylation Level		*RELA* Methylation Level	
		Mean (%)	*p*-Value	Mean (%)	*p*-Value
NF-κB p50 transcript	Negative	1.31 ± 0.87	0.717	1.33 ± 0.64	0.182
	Positive	1.41 ± 0.99		1.55 ± 0.48	
NFκB p50 transcript in TNF-α(+) tumor	Negative	1.42 ± 1.27	0.691	1.17 ± 0.53	0.002 *
	Positive	1.27 ± 0.85		1.71 ± 0.39	

* Indicates statistically significant (*p* < 0.05).

**Table 2:** Association of methylation levels of the *NFKB1* gene and the *RELA* gene with inflammatory markers in tumor tissues.

Inflammatory Markers		*NFKB1* Methylation Level		*RELA* Methylation Level	
		Mean Level (%)	*p*-Value	Mean Level (%)	*p*-Value
TNF-α, *n* = 46	Negative	1.56 ± 0.92	0.055	1.18 ± 0.52	0.009 *
	Positive	1.06 ± 0.80		1.71 ± 0.39	
IL-4, *n* = 47	Negative	1.17 ± 1.02	0.082	1.47 ± 0.52	0.757
	Positive	1.60 ± 0.73		1.51 ± 0.52	
Intratumoral inflammation, *n* = 47	Negative	1.43 ± 1.03	0.350	1.40 ± 0.56	0.107
	Positive	1.19 ± 0.61		1.70 ± 0.44	
Peritumoral inflammation, *n* = 36	Negative	0.97 ± 0.87	0.139	1.32 ± 0.58	0.460
	Positive	1.55 ± 0.97		1.49 ± 0.58	

* Indicates statistically significant (*p* < 0.05).

**Table 3:** Association of NF-κB p50 transcript with inflammatory markers in tumor tissues.

Inflammatory Markers		NFκB p50 Transcript		
		Negative (%)	Positive (%)	*p*-Value
TNF-α, *n* = 47	Negative	55.2	44.8	0.001 *
	Positive	5.6	94.4	
IL-4, *n* =47	Negative	54.5	45.5	0.014 *
	Positive	20.0	80.0	
Intratumoral inflammation, *n* = 47	Negative	33.3	66.7	0.493
	Positive	45.5	54.5	
Peritumoral inflammation, *n* = 36	Negative	25.0	75.0	0.682
	Positive	39.3	60.7	

* Indicates statistically significant (*p* < 0.05).

**Table 4:** Association of methylation levels of the *NFKB1* gene and *RELA* gene with clinicopathologic characteristics in tumor tissues (*n* = 47).

Clinicopathologic Variables		*NFKB1* Methylation Level		*RELA* Methylation Level	
		Mean Level (%)	*p*-Value	Mean Level (%)	*p*-Value
Age (years)	≤50	1.70 ± 0.77	0.062	1.47 ± 0.50	0.960
	>50	1.17 ± 1.00		1.47 ± 0.58	
Menopausal status	Premenopause	0.84 ± 0.98	0.208	1.63 ± 0.17	0.514
	Postmenopause	1.49 ± 0.96		1.43 ± 0.60	
Tumor size (cm)	≤2	1.30 ± 0.89	0.505	1.41 ± 0.53	0.405
	>2	1.49 ± 1.03		1.55 ± 0.57	
Node Metastasis	Negative	1.30 ± 0.92	0.463	1.46 ± 0.58	0.830
	Positive	1.51 ± 0.99		1.49 ± 0.51	
Histologic grade	I	1.78 ± 0.91	0.386	1.28 ± 0.17	0.255
	II	1.20 ± 1.33		1.67 ± 0.34	
	III	1.33 ± 0.72		1.47 ± 0.55	
Stage	I	1.35 ± 0.98	0.982	1.42 ± 0.48	0.598
	II	1.39 ± 0.95		1.57 ± 0.52	
	III	1.30 ± 1.24		1.34 ± 1.18	
Molecular subtype	Luminal A	0.93 ± 0.79	0.392	1.47 ± 0.61	0.371
	Luminal B	1.55 ± 1.04		1.48 ± 0.54	
	HER2	1.376 ± 1.22		1.29 ± 0.58	
	Basal-like	1.80 ± 0.35		2.00 ± 0.69	
	Positive	1.33 ± 1.00		1.51 ± 0.56	
ER status	Negative	1.45 ± 0.78	0.723	1.54 ± 0.53	0.723
	Positive	1.34 ± 1.02		1.44 ± 0.56	
PR status	Negative	1.53 ± 0.95	0.573	1.54 ± 0.64	0.650
	Positive	1.33 ± 0.95		1.45 ± 0.53	
HER2 overexpression	Negative	1.20 ± 0.88	0.155	1.58 ± 0.60	0.238
	Positive	1.65 ± 1.08		1.67 ± 0.52	
Ki-67	<14%	1.27 ± 0.69	0.761	1.65 ± 0.27	0.351
	≥14%	1.39 ± 0.99		1.44 ± 0.58	

HER2, human epidermal growth factor 2; ER, estrogen receptor; PR, progesterone.
